# Management of Leak After Revision of Roux-en-Y Gastric Bypass for Weight Regain by Internal Pigtail Drainage

**DOI:** 10.1007/s11695-023-06731-y

**Published:** 2023-07-19

**Authors:** Mohamed Hany, Mohamed Samir, Mohamed Ibrahim, Ahmed Zidan, Ann Samy Shafiq Agayby, Anwar Ashraf Abouelnasr, Bart Torensma

**Affiliations:** 1 Department of Surgery, Medical Research Institute, Alexandria University, 165 Horreya Avenue, Hadara, Alexandria, 21561 Egypt; 2 Madina Women’s Hospital, Alexandria University, Alexandria, Egypt; 3 Leiden University Medical Center (LUMC), Leiden, The Netherlands

## Introduction

Patients who undergo bariatric metabolic surgery (BMS) may experience difficulties in achieving their weight loss goals, regain weight, or develop complications related to surgery, with a prevalence rate of 40%. Revisional BMS is necessary in 25% of cases within 10 years [1, 2]. This multimedia article discusses the management of a leak following the revision of Roux-en-Y gastric bypass (RYGB) surgery for weight regain. After stent placement, the stent subsequently migrated, exposing the fistula site. A double pigtail tube was placed to drain the fistula, and the patient completely recovered. This article provides an overview of the pre and post-workup plan and findings.

## Presentation and Pre-workup

A 40-year-old female patient underwent RYGB at an external center in 2015 with no available operative data. The patient presented with weight regain and requested revisional surgery. Although the patient had lost 45 kg in the first year after the initial surgery, by August 2022, her weight had increased to 131 kg, with a BMI of 49 kg/m^2^. The patient reported no gastrointestinal symptoms, good food tolerance, adherence to a multivitamin regimen, and normal laboratory findings.

Several findings were identified following a multidisciplinary team consultation involving a surgeon, nutritionist, psychiatrist, and preoperative workup, including imaging and endoscopy. These included (1) non-adherence to follow-up visits, consumption of a high-calorie diet, lack of physical exercise, and mindless eating, (2) menstrual irregularities and osteoarthritis, (3) a computed tomography (CT) scan revealing a stomach volume of 490 ml and a prior cholecystectomy, and (4) an upper gastrointestinal endoscopy showed RYGB configuration with a long pouch, without a candy cane or hiatal hernia. Based on the intraoperative findings, the patient consented to redo RYGB surgery with pouch resizing and distalization (lengthening of the biliary limb). Before the surgery, the patient lost 6 kg over 6 weeks through dietary intervention and dialectical behavioral therapy.

## Revision Surgery

Revision surgery was performed, which included adhesiolysis, gastric pouch, and gastrojejunal anastomosis resizing over a calibration tube. Additionally, the anastomosis was distalized. The patient’s original total bowel length was 700 cm, consisting of a biliary limb (BL) of 100 cm, an alimentary limb (AL) of 150 cm, and a common channel (CC) of 450 cm. The AL at the jejunojejunostomy was divided and relocated 150 cm distally to create a total alimentary limb length (TALL) of 445 cm, ensuring a 300 cm CC. The BL was lengthened to 250 cm. All stable lines were reinforced with absorbable PDS V lock 3.0 (Covidien, Mansfield, MA, USA) sutures, and a drain was inserted. The patient was hemodynamically stable, tolerated oral fluids, and was discharged on the second postoperative day following drain removal.

## Postoperatively

The patient presented with fever, epigastric pain, and left shoulder pain 1 week after the surgery. The pulse rate was approximately 105 beats/min, and a CT scan with oral contrast revealed scattered foci of pneumoperitoneum near the esophagogastric junction with contrast extravasation. The patient was started on total parenteral nutrition and placed on nothing by the mouth while being treated with intravenous antibiotics. Laboratory findings indicated leukocytosis and elevated C-reactive protein. The fistula protocol was implemented, and conservative management was decided upon as the collection was confined to the surgical bed. Upper gastrointestinal imaging revealed a small fistula in the upper part of the gastric pouch, prompting the placement of a covered self-expanding metal stent (SEMS) to cover the fistula (Niti-S MEGA Esophageal Stent, Taewoong Medical, Gyeonggi-do, South Korea). The stent allows for prompt oral intake and reduced tension within the pouch, facilitating the closure of the fistula.

External pigtail tube insertion was planned; nevertheless, during the insertion of the external pigtail under CT guidance, the stent migrated, and retrieval with an endoscope failed, resulting in the exposure of the fistula. An endoscopic internal drainage (EID) using internal/intraluminal drains (double pigtail) was placed in this situation. The patient tolerated the nasojejunal feeding tube inserted through the jejunum well. The stent’s position was followed with abdominal CTs, and it was found to stop before enteroenteric anastomosis. The decision was made to extract it laparoscopically.

The external drain was subsequently removed, and the nasojejunal tube was retained for an additional week, while the patient gradually transitioned to oral fluids and a soft diet. The nasojejunal tube was removed, and the patient was permitted to continue an oral diet. The patient was discharged 1 month after the second admission, the internal pigtail was removed 3 weeks afterward, and a follow-up CT performed 2 months later showed no significant findings.

## Discussion

Revisional BMS carries a higher complication rate because of the added technical difficulty and patient-associated medical problems [1]. Several options for treating leaks are possible, from conservative treatment with fistula management to a more comprehensive technique with exploration surgery [3–5].

Recent systematic reviews showed that SEMSs are safe and used to treat GI leaks after BMS, whereby the stents showed success in 80–94% of acute anastomotic leaks. Regarding complications, the SEMS showed reflux, chest pain, gastric ulcer, bleeding, nausea, or vomiting. While pain (6–7%), ulcer (4%), vomiting (11%), perforation (1.85%), and bleeding (1.0%) were relatively uncommon. On migrations, the overall proportion in BMS was 23% (in RYGB 30.6% and LSG 28.0%) [3–5].

When distalazation was performed, the total alimentary limb length (TALL) and common channel (CC) length were the main variables for weight loss and minimizing nutritional deficiencies. A 2022 systematic review of 21 studies found that shortening TALL and therefore increasing the length of the BL significantly affect weight loss outcomes. Only two studies did not find extra weight loss, but this was because the BL was increased from 50 to 75 cm, and one study was limited because of a small sample size (*n* = 16). A TALL of less than 400 cm and CC of less than 200 cm should be avoided to prevent severe protein malnutrition and still maintain the best weight loss. The reported incidence of protein malnutrition ranges from 3.4 to 63.6% for patients who underwent limb-lengthening procedures. In our case, a TALL of 445 cm and CC of 300 cm were used, which falls within the suggested limits [6]. After this case report, the discussion remains on whether SEMS suits patients with an RYGB, despite the same migration ratio. The sleeve in the LSG has a closed environment because of the pylorus and duodenum; this can help to prevent the SEMS from moving further.

In 2022, a systematic review was conducted on endoscopic internal drainage (EID) treatment in BMS, which included ten studies published from 2015 to 2020. The overall success rate for EID treatment was 91.6% for leak closure, with a median treatment duration of 78.4 days (50.1–106.7). Complications associated with EID were reported in four studies and included stenoses (*n* = 8), perforation (*n* = 1), esophageal ulceration (*n* = 3), bleeding (*n* = 2), and splenic hematoma (*n* = 1). All studies together only included 232 patients, of which 80% underwent LSG and 20% underwent RYGB [7].

The literature after 2020 showed 25 studies, of which eight focused on EID and leakage therapy in BMS. Among these eight studies, five included only LSG patients, one included only OAGB patients, and two included only 13.4% and 18.8% of RYGB patients. The median (min-max) number of patients included in the studies was 39 (range: 5–1020). Despite the presence of EID in the literature, only 18 studies have been conducted from 2015 until now, with a main focus on LSG and the low power of included patients.

## Conclusion

Treatment plans should be tailored and adapted to the individual patient’s unique condition and the underlying cause of the leak. Metal stent insertion should not be commonly used after RYGB due to migration risk and anatomical changes. Alternatively, combining a double pigtail stent and external percutaneous drainage may be a safe and effective first-line approach for stable patients with local sepsis.

## Supplementary Information

ESM 1 (MP4 131524 kb)
